# Effects of long-term cigarette smoke exposure on bone metabolism, structure, and quality in a mouse model of emphysema

**DOI:** 10.1371/journal.pone.0191611

**Published:** 2018-01-30

**Authors:** Mamoru Sasaki, Shotaro Chubachi, Naofumi Kameyama, Minako Sato, Mizuha Haraguchi, Masaki Miyazaki, Saeko Takahashi, Takayoshi Nakano, Yukiko Kuroda, Tomoko Betsuyaku, Koichi Matsuo

**Affiliations:** 1 Division of Pulmonary Medicine, Keio University School of Medicine, Tokyo, Japan; 2 Division of Materials and Manufacturing Science, Graduate School of Engineering, Osaka University, Suita, Japan; 3 Laboratory of Cell and Tissue Biology, Keio University School of Medicine, Tokyo, Japan; University of California Davis, UNITED STATES

## Abstract

Smoking is a common risk factor for both chronic obstructive pulmonary disease (COPD) and osteoporosis. In patients with COPD, severe emphysema is a risk factor for vertebral fracture; however, the effects of smoking or emphysema on bone health remain largely unknown. We report bone deterioration in a mouse model of emphysema induced by nose-only cigarette smoke (CS) exposure. Unexpectedly, short-term exposure for 4-weeks decreased bone turnover and increased bone volume in mice. However, prolonged exposure for 20- and 40-weeks reversed the effects from suppression to promotion of bone resorption. This long-term CS exposure increased osteoclast number and impaired bone growth, while it increased bone volume. Strikingly, long-term CS exposure deteriorated bone quality of the lumbar vertebrae as illustrated by disorientation of collagen fibers and the biological apatite c-axis. This animal model may provide a better understanding of the mechanisms underlying the deterioration of bone quality in pulmonary emphysema caused by smoking.

## Introduction

There is ample evidence in the literature that smoking is a risk factor for low bone mineral density (BMD) and osteoporotic fracture [1]. Smoking is also a risk factor for chronic obstructive pulmonary disease (COPD), which is a highly prevalent condition that causes significant morbidity and mortality and is commonly associated with many extra-pulmonary abnormalities such as cardiovascular disease, skeletal muscle wasting, and osteoporosis [2–4]. However, the pathophysiologic mechanisms underlying osteoporosis in cigarette smokers have not been fully explored.

Bone is a dynamic organ in which osteoclasts resorb old bone and osteoblasts form new bone. These two types of cells are regulated by systemic and local factors including estrogen, vitamin D metabolites, parathyroid hormone, receptor activator of nuclear factor-κB ligand (RANKL), and numerous other molecules produced by the cells themselves [5]. Alterations in bone metabolism may occur as indirect or direct effects of nicotine or other constituents of cigarette smoke (CS) on osteoblastic bone formation and osteoclastic bone resorption [6]. In culture, low levels of nicotine increase osteoblast differentiation and enhance the mRNA expression of osteocalcin, type I collagen and alkaline phosphatase [7]. Nicotine was also shown to suppress cathepsin K (*Ctsk*) and matrix metalloproteinase 9 (*Mmp9*) expression in osteoclasts, and bone resorption by RANKL-stimulated mouse RAW 264.7 cells [8].

Chronic whole-body CS exposure for 12 weeks in mice induced long bone osteoporotic changes such as decreases in stiffness and bone weight [9], while no significant loss in vertebral trabecular bone volume was observed [10]. In addition, CS exposure decreased bone formation and increased bone resorption in 4-month smoke-exposed female rats [11]; however, special caution should be taken in interpreting these data because whole-body passive smoke may not be an appropriate model of active smokers or COPD in humans [12]. The effects of CS on bones are thought to be associated with the routes and duration of CS exposure [13]. Recently, Cielen *et al*. (2016) reported that smoking (24 weeks) via a nose-only exposure system caused pulmonary emphysema and reduced food intake with a subsequent loss of body weight as well as fat and lean muscle mass, but increased the trabecular bone volume of the tibia [14]. In animal models, the effects of CS exposure on bone are controversial.

In addition, many studies have demonstrated the limited capacity of using BMD alone to determine the mechanical properties of bone [15, 16]. The direction of the c-axis of the biological apatite crystallite is parallel to the collagen fibers. Therefore, the degree of c-axis orientation is expected to be a bone quality parameter that determines bone strength, and shows a more significant contribution to mechanical properties than BMD in regenerated defected long bone [17, 18].

In this study, a nose-only smoking mouse model was applied, as previously established by our group [19], in which mice developed emphysema, a pulmonary phenotype of human COPD, after 3 months of CS exposure. Using this mouse model, we evaluated the short-term (4 weeks) and long-term (20 and 40 weeks) effects of CS exposure on the metabolism, structure, and quality of the vertebral column.

## Materials and methods

### Mice

C57BL/6J (B6) mice (9–10 weeks old) were purchased from Oriental Yeast (Tokyo, Japan). Mice were housed in plastic cages under a 12:12 h light-dark cycle, fed standard chow (CE-2, Nihon CLEA, Tokyo, Japan), and given free access to food and water. Animal experiments were performed in accordance with the Institutional Guidelines on Animal Experimentation at Keio University. The protocols were approved by the Keio University Institutional Animal Care and Use Committee (Approval No: 12019).

### CS exposure

Mice were exposed to mainstream CS generated from commercially available filtered cigarettes (Marlboro, 12 mg tar/1.0 mg nicotine) and inhaled CS through their noses as previously reported by our group [19]. Briefly, an apparatus for the CS inhalation (SIS-CS system, Shibata Scientific Technology Ltd., Tokyo, Japan), consisting of both a CS generator (SG-300) and an inhalation chamber to which 20 body holders were set at a time, was used. Fresh cigarettes purchased within 1 month of the study were used throughout the experiments. The CS was generated at a stroke volume of 15 mL and 10 puffs/min, and was diluted with compressed air in which the mass concentration of total particulate matter was 1,202 ± 196 mg/m^3^.

Ovariectomized B6 female mice (B6-OVX) were 9–10 weeks old at the time of surgery and exsanguinated under carbon dioxide narcosis 4 weeks after surgery [20]. We confirmed that uterine weights/body weights (%) were significantly decreased 4 weeks after ovariectomy (0.64±0.17, B6 sham-operated females, *n* = 10 vs 0.09±0.03, B6-OVX, *n* = 9, p<0.05, *t*-test).

Mice were exposed to CS for 60 min/day and 5 days/week for up to 4, 20, and 40 weeks. Age- and sex matched air-exposed control mice were exposed over the same time period.

### Micro-CT

The X-ray micro computed tomography (micro-CT) system (R_mCT2, Rigaku, Tokyo, Japan) was operated with the following parameters: 90 kV, 160 μA, abdominal CT; standard mode, 26 sec, 30 x 30 mm field of view (FOV) (60 x 60 μm pixel size), bone CT; fine mode, 3 min, 5 x 5 mm FOV (10 x 10 μm pixel size). Mice were scanned in the prone position with inhalation anesthesia of mixed isoflurane (Pfizer Japan Inc., Tokyo, Japan) and oxygen through a nose cone. Fat volume analysis was evaluated at the third lumbar, level, discriminating between subcutaneous and visceral fat using body composition analysis software (Metabolic analysis, Rigaku, Tokyo, Japan). Bone structure was evaluated in fourth lumbar vertebrae using bone analysis software (3D-Bon, RATOC, Tokyo, Japan). For each bone, vertebral body height, cortical cross sectional area, trabecular bone volume to tissue volume ratio (BV/TV), trabecular number (Tb.N), trabecular thickness (Tb.Th), and trabecular spacing (Tb.Sp) were measured.

### Bone histomorphometry

The mice were exsanguinated by severing the abdominal aorta under carbon dioxide narcosis. The fourth lumbar vertebra was fixed in 4% paraformaldehyde, decalcified in 10% ethylenediaminetetraacetic acid (pH 7.2), and embedded in paraffin. Sections of 5 μm thickness were cut and stained with hematoxylin and eosin (H&E). The region of interest was defined as a 100 mm^2^ area in the center of the vertebral body, excluding cortical regions and growth plates. Osteomeasure software (Osteomeasure, OsteoMetrics, Decatur, GA, USA) was used to quantify the bone surface (BS), osteoclast surface (Oc.S), and osteoblast surface (Ob.S). Then osteoclast surface/bone surface (Oc.S/BS) and osteoblast surface/bone surface (Ob.S/BS) were calculated.

### Mechanical testing

Fifth or sixth lumbar vertebrae was placed in the materials testing machine (TK-252C, Muromachi Kikai Co., Tokyo, Japan) between two parallel plates and was compressed at a constant velocity of 2.5 mm/min until failure. The stiffness (N/mm), ultimate compressive load (N), and energy-to-failure (mJ) were calculated as the mechanical properties from the load-displacement curve [21] using re-analysis software (RAS-252C, Muromachi Kikai Co.).

### Histologic analysis of collagen fibers in bone

Analysis of spatially aligned collagen fibers was performed in the paraffin sections with a microscope (BX53, Olympus, Tokyo, Japan) using a polarizing lens (U-TAD & U-POT, Olympus). Bright shining of polarized light qualitatively revealed the parallelism of collagen fibers in tissue sections [22].

### C-axis measurement

The degree of directionality of the c-axis in the biological apatite crystals was determined by X-ray diffraction analysis using a microbeam X-ray diffraction (μXRD) system as the relative intensity ratio of the (002) diffraction peak to the (310) peak in the X-ray profile. The intensity ratio of (002)/(310) increases with an increase in the degree of preferential orientation of the c-axis of the biological apatite [18].

### Biochemical analysis

Spot urine was collected in the morning, and blood was collected by abdominal aorta puncture after mice were sacrificed under carbon dioxide narcosis. The urine and serum samples were stored at -80°C until use. Urinary deoxypyridinoline (DPD), a marker of bone resorption, and creatinine (Cr) concentrations were measured using the EIA Kit (Osteolinks-DPD, Sumitomo, Osaka, Japan) and the enzyme test (Mitsubishi Kagaku, Tokyo, Japan), respectively. Urinary DPD was corrected for urinary Cr concentration. Serum levels of osteocalcin, a marker of bone formation, were measured using the EIA Kit (Mouse Osteocalcin ELISA kit, Biomedical Technologies, Redwood City, CA, USA).

### Statistical analysis

Data are expressed as mean ± standard deviation (SD) and were analyzed by Student’s *t-*test. When we compared between air- and CS exposure in both B6-female and B6-OVX mice, data were analyzed with Student’s *t-*test. P values less than 0.05 were considered statistically significant. All of the data were analyzed using the JMP data analysis software for Windows, version 11.0.0 (SAS Institute Inc., Cary, NC, USA).

## Results

### Short-term CS exposure suppresses bone turnover and increases bone volume in female mice

**Fig 1:**
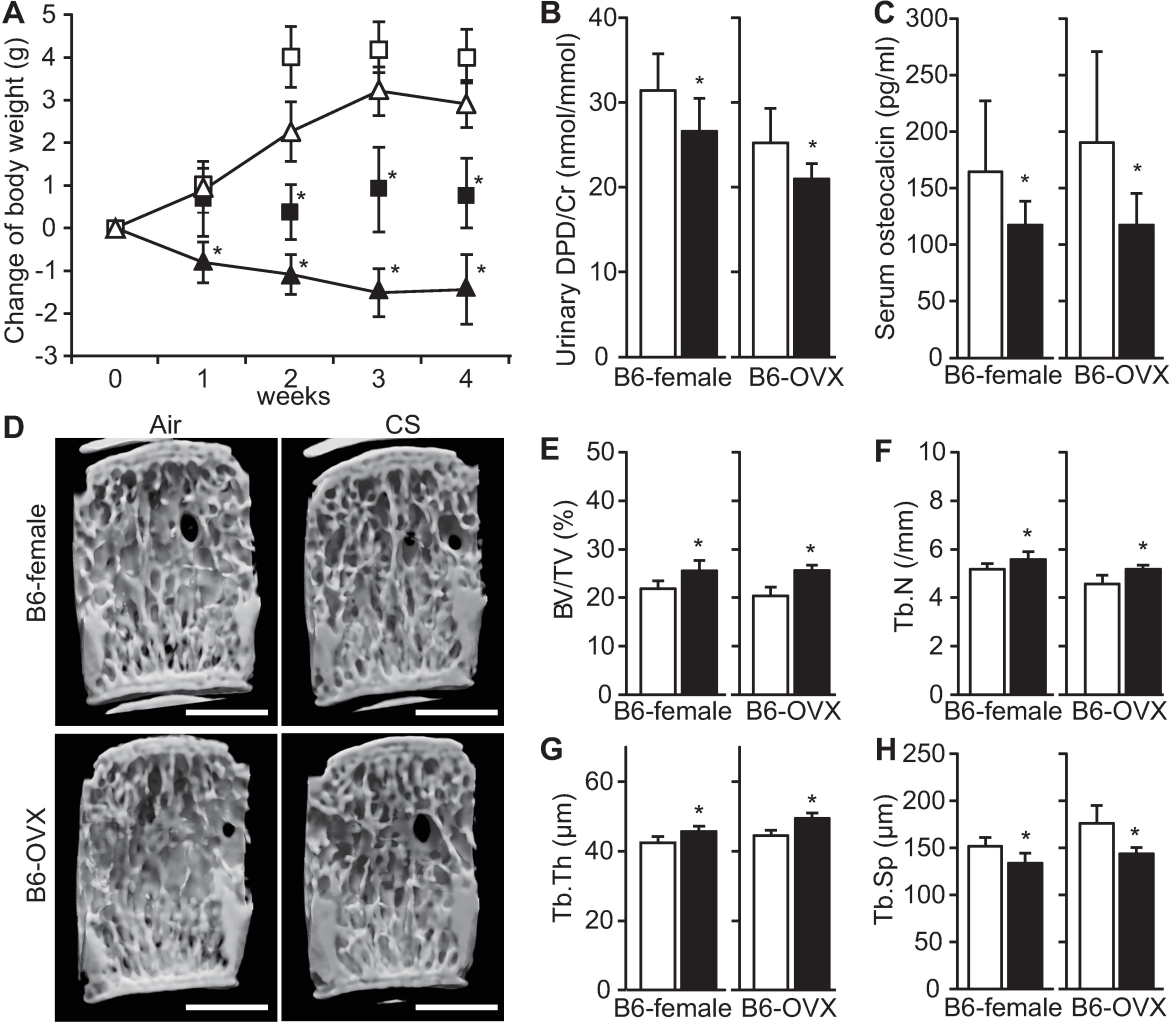
Changes in body weight, bone turnover, and bone structure after short-term CS exposure in mice with and without ovariectomy. (A) Longitudinal changes in body weight of C57BL/6J female (B6-female) mice over 4 weeks of air-exposure (open triangle, *n* = 10) or CS-exposure (closed, *n* = 9), and ovariectomized (B6-OVX) mice, of air-exposure (open square, *n* = 9) or CS-exposure (closed, *n* = 9). The body weight data are shown as means ± SDs. (B, C) Bone metabolism markers (urinary DPD and serum osteocalcin) after 4 weeks of air- (open bar) and CS exposure (closed bar). (D) Representative micro-CT 3D images of fourth vertebral body sections created by longitudinal cutting (left-oblique view). Scale bars = 1 mm. (E–H) micro CT bone structure analyses (BV/TV, Tb.N, Tb.Th and Tb.Sp) after 4 weeks of air- (open bar) and CS exposure (closed bar). The data are shown as means ± SDs. *P<0.05 between air- and CS-exposed mice. Statistical analyses were performed with Student’s *t*-test.

We examined the effects of short-term CS exposure using both B6 female mice (B6-female) and ovariectomized (B6-OVX) mice, which is a widely used model for estrogen deficiency. To examine the systemic effects of CS exposure to mice, body weight was monitored weekly. In B6-female mice, the body weight gradually increased, while CS-exposed B6-female mice began to lose weight soon after CS exposure. Similarly, in B6-OVX mice, the body weight increased prominently, while CS exposure attenuated the weight gain (Fig 1A). In contrast to our initial hypothesis that CS exposure would facilitate further bone resorption in those mice, the bone resorption marker urinary DPD and the bone formation marker osteocalcin were significantly decreased after 4 weeks of CS exposure compared with the respective air-exposed control mice in both B6-female and B6-OVX mice (Fig 1B and 1C).

We next examined vertebrae, because, in COPD patients, the risk of vertebral fractures was related to the severity of the disease [23]. In addition, B6-female and B6-OVX mice exhibited more trabecular bone volume in vertebral bodies following CS exposure for 4 weeks than their respective air-exposed control mice (Fig 1D), as demonstrated by the increase in BV/TV, Tb.N and Tb.Th, and the decrease in Tb.Sp on micro-CT compared with their respective air-exposed control mice regardless of ovariectomy status (Fig 1E–1H). To determine whether the observed bone phenotypes following CS exposure in female mice are reproducible in male mice, we performed short-term CS exposure in B6-male mice. We observed the same tendency in B6-male mice, suggesting that the increase in trabecular bone after short-term CS exposure was sex independent (S1 Fig).

### Effects of transition to long-term CS exposure on body weight and visceral fat volume

**Fig 2:**
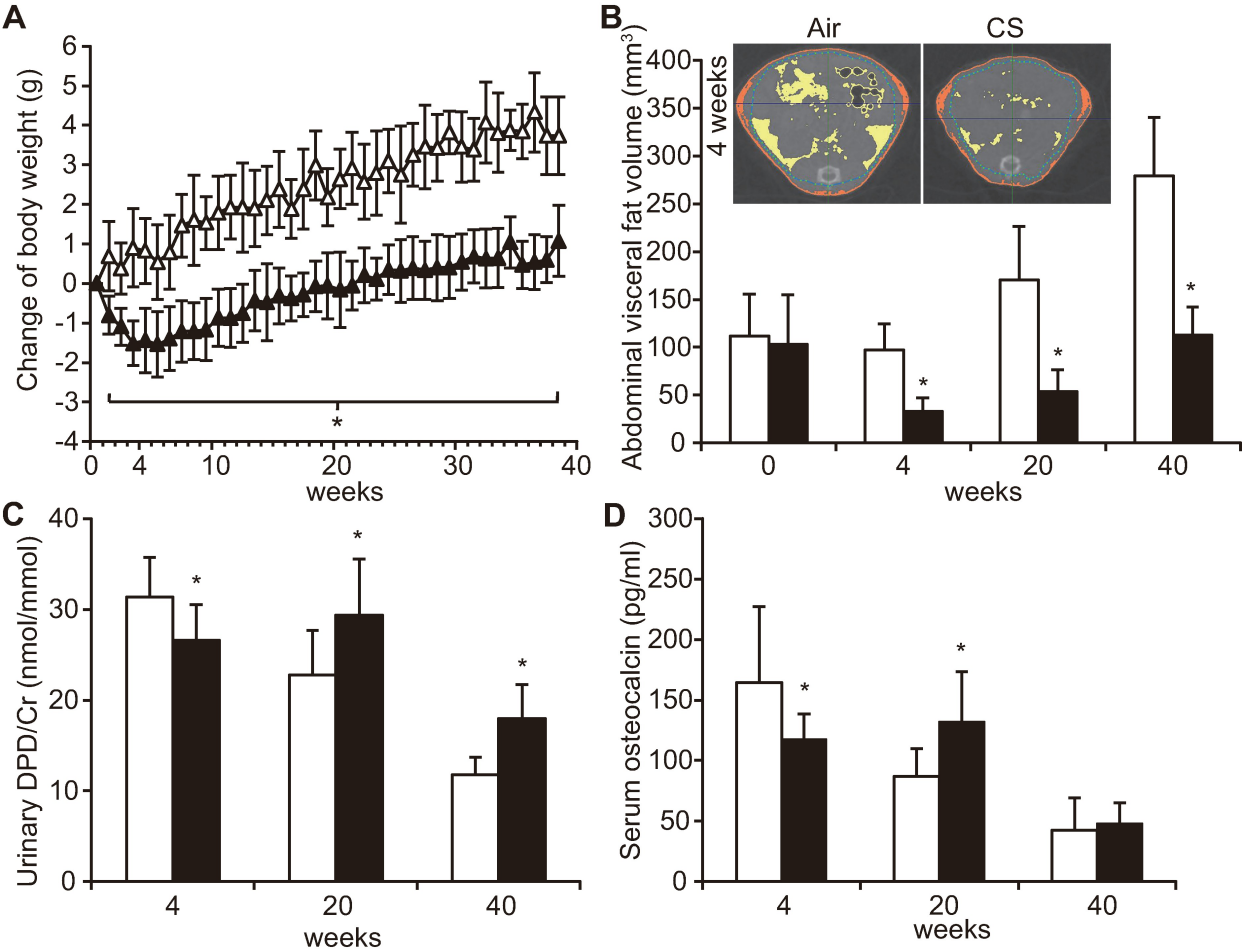
Changes of body weight, abdominal fat volume, and bone metabolism markers upon long-term CS exposure. (A) Longitudinal changes in body weight of air- exposed (open triangle: *n* = 5) and CS-exposed mice (closed triangle: *n* = 10 at the start, *n* = 9 at the end). The body weight data are shown as means ± SDs. *P<0.05 between air-exposed controls and CS-exposed B6-female mice. Statistical analysis was performed with Student’s *t*-test at each time point. (B) Representative micro-CT images (transverse view) of abdominal fat after 4 weeks of CS exposure distinguishing between visceral fat (yellow) and subcutaneous fat (orange). Abdominal visceral fat volume at 0, 4, 20, and 40 weeks of CS exposure. (C, D) Bone metabolism markers (urinary DPD and serum osteocalcin) at 4, 20, and 40 weeks of CS exposure. The data are shown as means ± SDs. *P<0.05 between air-exposed controls (open bars: *n* = 10 at 4 weeks, *n* = 10 at 20 weeks, *n* = 5 at 40 weeks) and CS-exposed B6-female mice (closed bars: *n* = 9 at 4 week, *n* = 5 at 20 weeks, *n* = 9 at 40 weeks). Statistical analysis was performed with Student’s *t*-test.

B6-female mice underwent repeated long-term exposure to CS up to 40 weeks, according to an established protocol [19]. There was no difference in daily energy intake between CS- and air-exposed groups at each time point. Body weight changes in CS-exposed mice were significantly different from air-exposed control mice during the 40 weeks (Fig 2A). CS-exposed mice lost up to about 6% of their weight until 4 weeks, after which they began gaining weight. This time-course trend was consistent with changes in visceral fat volume in the CS-exposed mice (Fig 2B). These findings suggest that at approximately 4 weeks, the systemic effects of CS exposure cause an initial decrease but subsequent increase in body weight and body fat.

### Prolonged CS exposure causes a change in suppression to promotion of bone resorption

As shown above, 4 weeks of CS exposure decreased both urinary DPD and serum levels of osteocalcin in B6 mice. However, after CS exposure up to 20 weeks, there were significantly higher urinary DPD and serum osteocalcin levels in CS-exposed B6-female mice compared with air-exposed control mice; this difference remained in urinary DPD at 40 weeks (Fig 2C and 2D). These data suggest that CS exposure suppresses both bone resorption and formation in the short term, but promotes bone resorption after 20 weeks.

### Long-term CS exposure increases the number of differentiated osteoclasts in lumbar vertebrae

**Fig 3:**
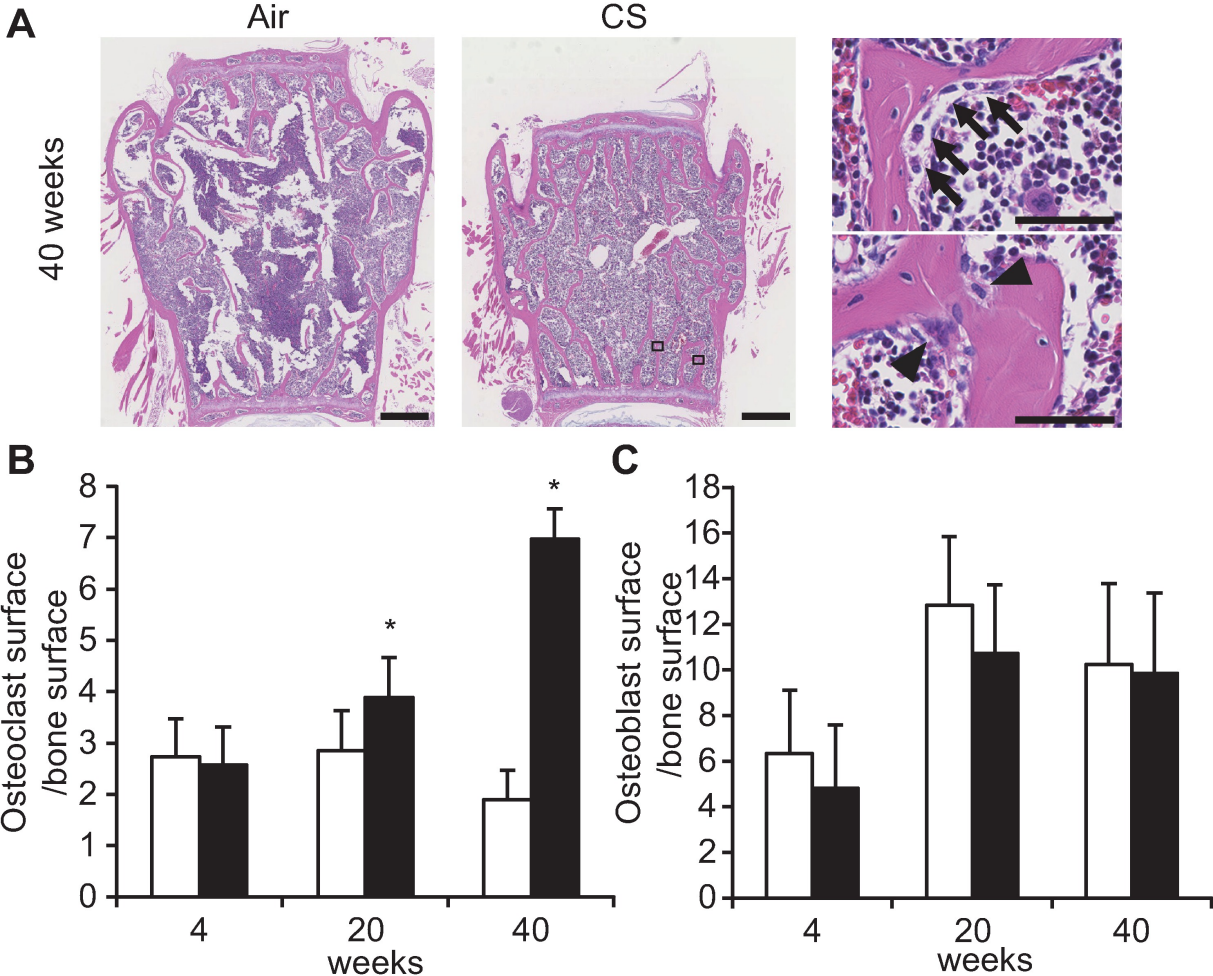
Effects of CS exposure on osteoclasts and osteoblasts of vertebra. (A) Representative H&E staining of the fourth vertebral body (longitudinal section) at 40 weeks in CS- and air-exposed mice. Representative osteoblasts (arrows) and osteoclasts (arrowheads) in the boxed areas are shown on the right at a higher magnification. Scale bars at low-power and high-power magnification are 500 and 50 μm, respectively. (B, C) Osteoclast surface/bone surface (Oc.S/BS) and osteoblast surface/bone surface (Ob.S/BS) after 4, 20, and 40 weeks of CS exposure. The data are shown as means ± SDs. *P<0.05 between air-exposed controls (open bars: *n* = 10 at 4 weeks, *n* = 10 at 20 weeks, *n* = 5 at 40 weeks) and CS-exposed B6-female mice (closed bars: *n* = 9 at 4 week, *n* = 5 at 20 weeks, *n* = 9 at 40 weeks). Statistical analysis was performed with Student’s *t*-test.

Bone histomorphometric changes were investigated in the fourth lumbar vertebral bodies of B6-female mice (Fig 3A). The Oc.S/BS was comparable at 4 weeks, and significantly increased in CS-exposed mice compared with air-exposed control mice at 20 and 40 weeks (Fig 3B). Those changes were consistent with the urinary DPD data (Fig 2C). On the other hand, no difference was observed in the Ob.S/BS between those two groups at any time point (Fig 3C).

### Long-term CS exposure impairs growth and increases the trabecular bone volume of lumbar vertebrae

**Fig 4:**
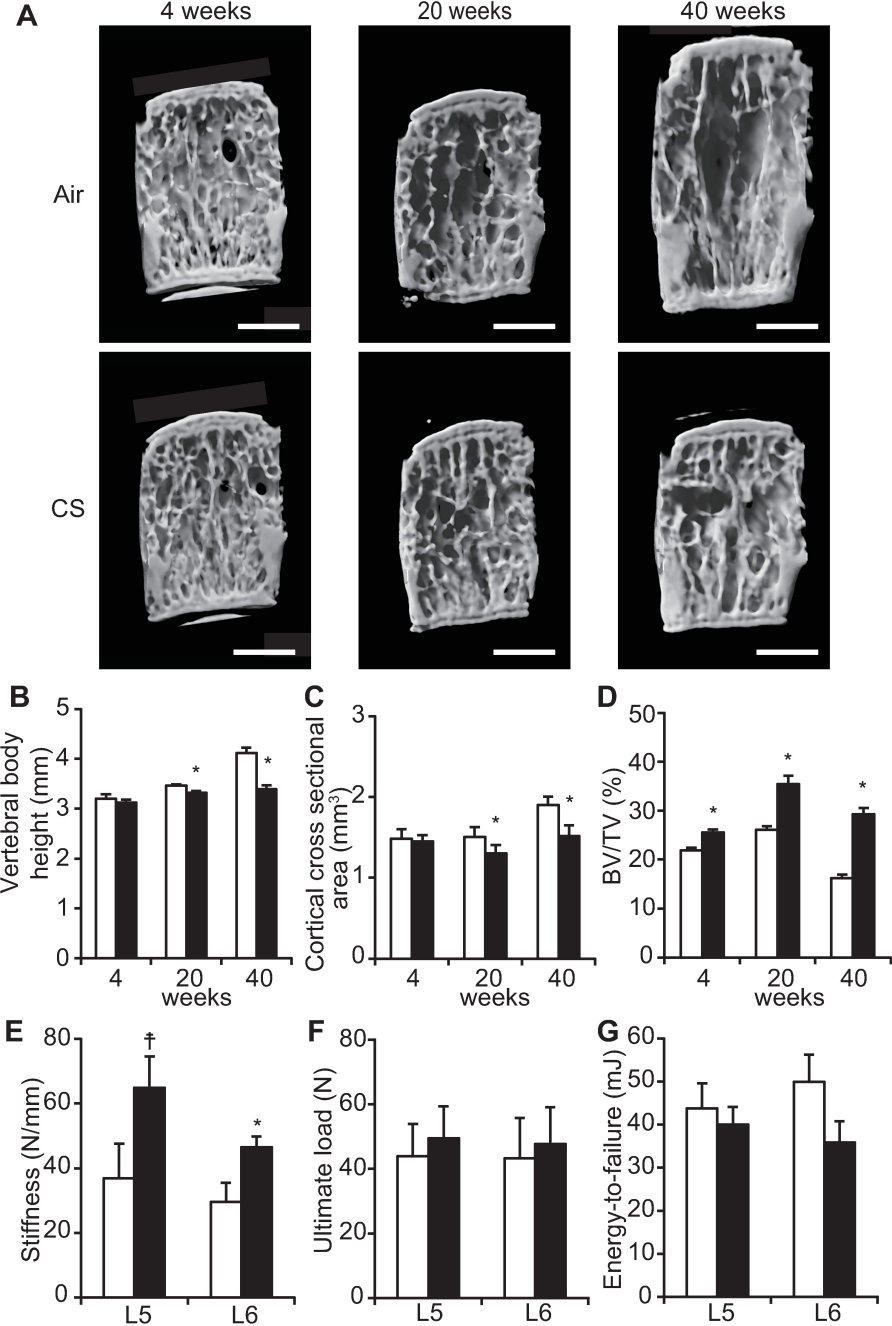
Effects of CS exposure on bone structure on micro-CT and mechanical strength tests of vertebral bodies. (A) At 4, 20, and 40 weeks of CS exposure, representative micro-CT 3D images of fourth vertebral body sections created by longitudinal cutting (left-oblique view). Scale bars = 1 mm. (B–D) Vertebral body height, cortical cross sectional area, and bone volume to tissue volume ratio (BV/TV). *P<0.05 between air-exposed controls (open bars: *n* = 10 at 4 weeks, *n* = 10 at 20 weeks, *n* = 5 at 40 weeks) and CS-exposed mice (closed bars: *n* = 9 at 4 week, *n* = 5 at 20 weeks, *n* = 9 at 40 weeks). (E–G) Stiffness, ultimate load and energy-to-failure of fifth and sixth lumbar vertebral bodies. The data are shown as means ± SDs. *P<0.05, ☨P = 0.07 between air-exposed controls (open bars, L5: *n* = 5, L6: *n* = 5) and CS-exposed mice (closed bars, L5: *n* = 9, L6: *n* = 9). The data are shown as means ± SDs. Statistical analysis was performed with Student’s *t*-test.

After 20 and 40 weeks of long-term CS exposure, changes in the bone size and structure of the trabecular bone were evaluated in the fourth lumbar vertebra using micro-CT (Fig 4A). Compared with air-exposed control mice, vertebral body height and cortical cross sectional area were significantly decreased in CS-exposed B6-female mice at 20 and 40 weeks (Fig 4B and 4C). These data indicate that long-term CS exposure impaired the growth of lumbar vertebral bodies. Conversely, BV/TV was elevated in CS-exposed mice compared with air-exposed control mice at each time point (Fig 4D). The cortical bone volume was no significant difference between CS and air-exposed mice. We performed mechanical strength tests (stiffness, ultimate load and energy-to-failure) of fifth and sixth lumbar vertebral bodies (L5 and L6) at 40 weeks of CS exposure. Stiffness of the vertebral body in CS-exposed mice was significantly increased at L6, although the difference did not reach statistical significance at L5 (Fig 4E). By contrast, ultimate load and energy-to-failure were not significantly changed in CS-exposed mice at 40 weeks compared with air-exposed control mice (Fig 4F and 4G), which could be attributed to the combined effects of decreased cortical cross sectional area and elevated trabecular bone volume. These data suggest that CS-induced increases in bone volume and stiffness in vertebral bodies did not increase bone mechanical strength measured, as determined by measuring ultimate load and energy-to-failure. In addition, the normal growth of vertebral bodies was retarded by long-term CS exposure.

### Long-term CS exposure deteriorates vertebral bone quality

**Fig 5:**
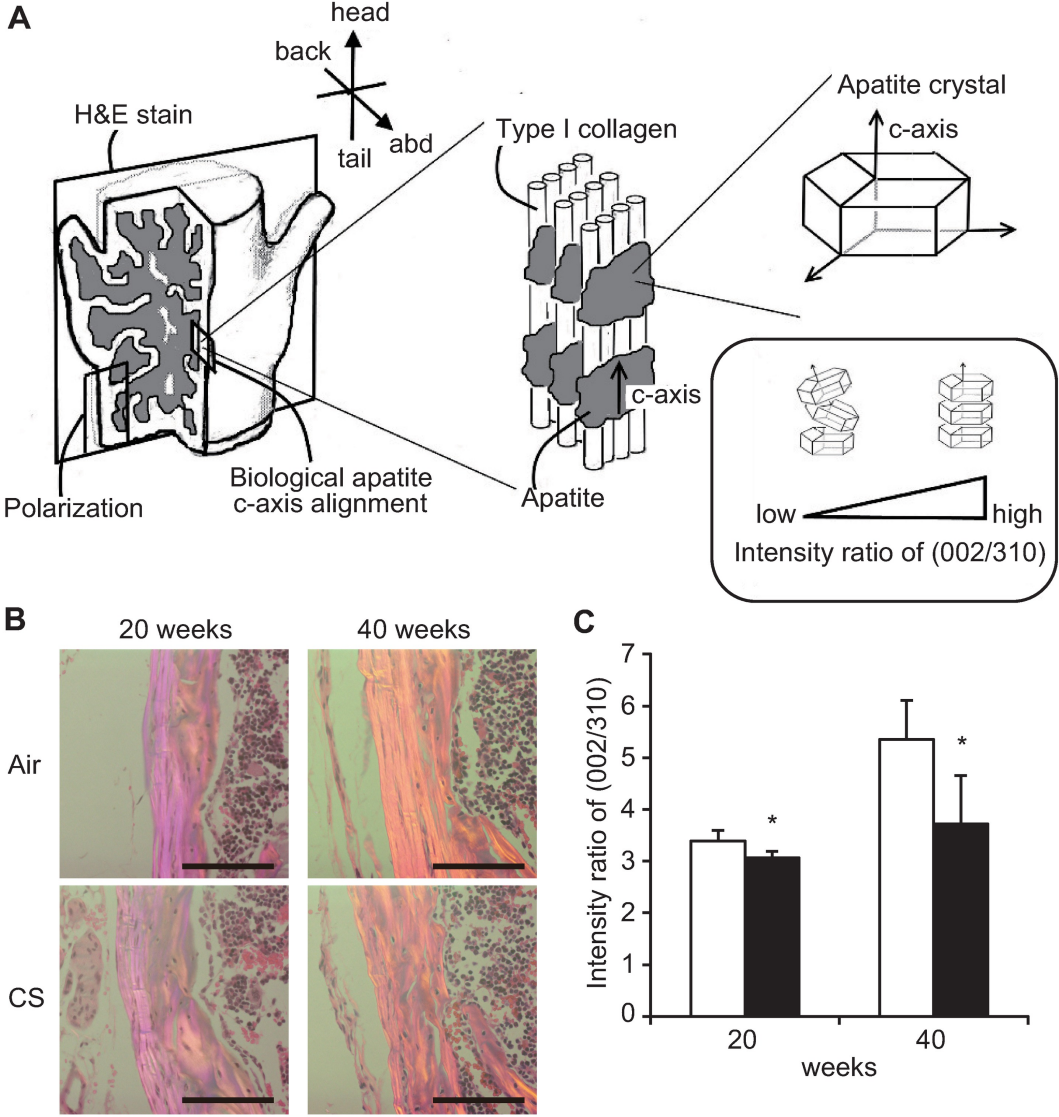
Effects of long-term CS exposure on collagen orientation and biological apatite c-axis alignment of vertebral bodies. (A) Schematic presentation of bone histological indices (H&E staining, polarization, collagen orientation and biological apatite *c*-axis alignment). (B) Representative polarizing microscope images in the fourth vertebral bodies (longitudinal section) of B6-female mice after 20 and 40 weeks of air- or CS exposure. Scale bars = 100 μm. (C) Intensity ratio of (002/310) as biological apatite c-axis alignment. The data are shown as means ± SDs. *P<0.05 between air-exposed controls (open bars: *n* = 10 at 20 weeks, *n* = 5 at 40 weeks) and CS-exposed B6-female mice (closed bars: *n* = 5 at 20 weeks, *n* = 12 at 40 weeks). Statistical analysis was performed with Student’s *t*-test.

To investigate the reason that bone mechanical strength did not increase despite the increase in bone volume and stiffness following long-term CS exposure, we evaluated collagen orientation and biological apatite c-axis alignment in vertebral bodies to determine bone quality (Fig 5A). Polarized light microscopy showed that the orientation of collagen fibers in CS-exposed B6-female mice was disturbed compared with that in air-exposed control mice at 20 and 40 weeks (Fig 5B). In addition, CS-exposed mice showed a significantly lower degree of preferential biological apatite c-axis alignment in the vertebral bodies at 20 and 40 weeks based on the relative intensity ratio of the (002) diffraction peak to the (310) peak in the X-ray profile (Fig 5C). These data further support the notion that long-term CS exposure deteriorates vertebral bone quality.

## Discussion

This is the first *in vivo* study to demonstrate that long-term CS exposure impairs the normal growth of lumbar vertebral bodies and deteriorates vertebral bone quality, as revealed by disorientation of collagen fibers and the biological apatite c-axis. Interestingly, however, short-term CS exposure decreased bone resorption and increased bone volume in mice.

**Fig 6:**
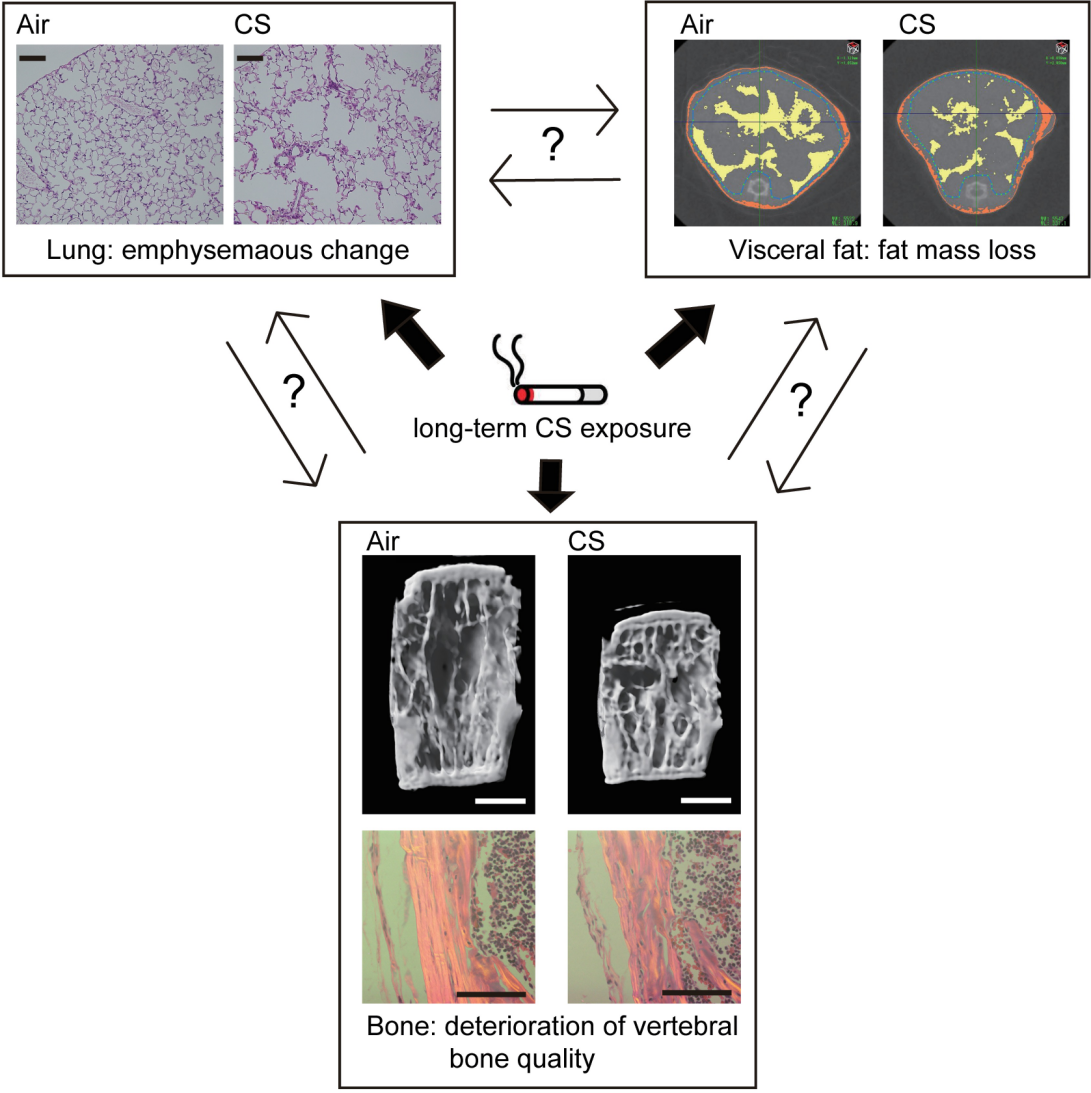
Effects of long-term CS exposure on lung, fat and bone. The association of deteriorating bone quality with low fat mass and presence of emphysema indicates that the underlying mechanisms link the lung to the skeletal system; however, whether this is a direct effect of long-term CS exposure or a secondary effect of pulmonary and extra-pulmonary changes remains unclear. Representative lung sections stained with H&E after 40 weeks of CS exposure (scale bars = 100 μm). Representative micro-CT images (transverse view) of abdominal fat after 40 weeks of CS exposure distinguishing between visceral fat (yellow) and subcutaneous fat (orange). Representative micro-CT 3D (scale bars = 1 mm) and polarizing microscope images (scale bars = 100 μm) of fourth vertebral body after 40 weeks CS exposure.

Collagen alignment affects bone strength independent of bone volume, and collagen alignment parameters correlated with bone stiffness in pre-diabetic and diabetic rats [24]. Our study suggests that CS-induced osteoporosis may change collagen orientation and biological apatite c-axis alignment without bone loss. Long-term CS exposure can lead to abnormalities in vertebral bodies with regard to bone metabolism, structure, and quality. Smoking is a common lifestyle risk factor for both COPD and bone loss, and osteoporosis is more prevalent in COPD patients than in healthy smokers [25]. A confounding factor influencing BMD is fat mass; this relationship was reported by examining the effects of body mass and adiposity on adult BMD. Larger adipose stores and increased body fat percentage lead to increased BMD [26]. Smokers generally have a lower body mass index (BMI) and decreased adipose tissue than nonsmokers, which may affect the BMD differences that have been reported in smokers and nonsmokers [27]. Recent evidence has suggested that female COPD patients with a low BMI and radiographic emphysema are at risk for low BMD and vertebral bone fracture [28, 29]. However, there have been few *in vivo* studies of the extra-pulmonary system in CS-induced COPD animal models [30]. In our study, CS-exposed B6-female mice showed drastic weight loss accompanied by visceral fat mass loss within 4 weeks of CS exposure. A study by Sasaki *et al*. (2015) showed that long-term CS exposure induced emphysematous change in the lungs of B6-female mice [19]. In our mouse model, the association of deteriorating bone quality with low BMI and presence of emphysema indicate that the underlying mechanisms link the lung to the skeletal system; however, whether this is a direct effect of CS exposure or a secondary effect of pulmonary and extra-pulmonary changes remains unclear (Fig 6).

In human studies, it has generally been reported that smoking decreases bone volume and BMD by enhancing bone resorption. In this study, however, we observed that CS exposure increased bone volume and BMD in mice. The direct suppression of osteoclastogenesis by CS could partly explain decreases in both resorption of the urinary bone resorption marker DPD and the bone formation marker serum osteocalcin in female mice after short-term CS exposure. The effects of nicotine on osteoclastogenesis may be related to the direct suppression of osteoclastogenesis by CS, which is in accordance with data from the study by Tanaka *et al*. [8]. In human studies, sex hormones, especially estrogen, are important bone anabolic factors; smoking can both decrease and increase estrogen levels [6]. However, estrogen is unlikely to be involved in the CS exposure-induced increase in bone volume and BMD, because not only B6-female mice, but also B6-OVX and B6-male mice showed decreased urinary DPD and osteocalcin levels after short-term CS exposure.

There were several limitations in this study. First, the mouse model used in this study does not reflect the human situation if we consider the age of the animals and duration of CS exposure, as we used relatively young animals that are still growing and gaining body weight. By contrast, most human studies are conducted in older population. Thus, a COPD animal model with older animals is worth testing. At present, we cannot exclude the possibility that the effects of CS exposure on bone metabolism and bone volume in mice might be similar to those in premenopausal, rather than postmenopausal women. Indeed, we ovariectomized 9–10 weeks old mice rather than older mice typically used in menopausal mice model [20]. The young age of the mice used would explain why osteoporotic changes in B6-OVX mice were marginal in this study, together with the fact that C57BL/6J mice are hard to loose bone mass upon ovariectomy compared with other strains [20]. Second, it is possible that the duration of CS exposure in this study was not enough. Indeed, after 20 weeks or longer of CS exposure, the markers of bone metabolism significantly shifted from a low to high turnover, in concordance with significant increases in osteoclast number in vertebral bodies. If the duration of CS exposure—generally less than 24 weeks CS [14], and currently 40 weeks—was extended, it is possible that an osteoporotic phenotype could be found. Patients with COPD have a much longer smoking history (of many years) and more pronounced systemic manifestations, including osteoporosis, as they age. Smoking history is a known risk factor for bone loss [27]; however, current smoking does not necessarily increase the risk of osteoporosis in COPD patients [29]. Third, total bone volume was increased with continuous smoking in our mouse model. This finding may be due to the fact that the mouse model of CS exposure only induced mild form of pulmonary emphysema [19]. Thus, the systemic effects of smoking in our mouse model may represent the early stage of extra-pulmonary manifestations in COPD patients.

To the best of our knowledge, this is the first study to demonstrate *in vivo* that long-term CS exposure suppresses normal growth and deteriorates vertebral bone quality, as illustrated by disorientation of collagen fibers and the biological apatite c-axis, even though 20 to 40 weeks of CS exposure increased bone volume. This animal model will substantially contribute to our understanding of the mechanisms underlying systemic manifestations with pulmonary emphysema in smokers, and will also contribute to efforts toward understanding the mechanisms and therapeutics associated with osteoporosis in COPD patients.

## Supporting information

S1 Fig Body weight, bone turnover, and bone structure of B6-male mice after short-term CS exposure. (A) Longitudinal changes in body weight of C57BL/6J male (B6-male) mice after 4 weeks exposure to air (open square, *n* = 5) or CS (closed square, *n* = 5). The body weight data are shown as means ± SDs. *P<0.05 between air- and CS-exposed mice in B6-male mice. Statistical analysis was performed with Student’s *t*-test at each time point. (B, C) Bone metabolism markers (urinary DPD and serum osteocalcin) after 4 weeks of CS exposure. (D) Representative micro-CT 3D images of fourth vertebral body sections that created by a longitudinal cutting (left-oblique view). Scale bars = 1 mm. (E–H) micro-CT bone structure analyses (BV/TV, Tb.N, Tb.Th and Tb.Sp) after 4 weeks of CS exposure. The data are shown as means ± SDs. *P<0.05 between air-exposed controls (open bars, *n* = 5) and CS-exposed mice (closed bars, *n* = 5). Statistical analysis was performed with Student’s *t*-test.
(EPS)
